# Reduced heart rate variability is associated with disease severity in AL amyloidosis: a retrospective cohort study

**DOI:** 10.3389/fmed.2026.1882210

**Published:** 2026-08-27

**Authors:** Xiao-hang Pei, Su-qiong Zuo, Pan Zhou, Rong-jun Ma, Zun-min Zhu, Xiao-li Yuan

**Affiliations:** Department of Hematology, Henan Provincial People’s Hospital, Zhengzhou, China

**Keywords:** AL amyloidosis, autonomic dysfunction, cardiac involvement, disease progression, heart rate variability, prognosis

## Abstract

**Background:**

Autonomic nervous system involvement is common in systemic amyloidosis, yet the clinical relevance of heart rate variability (HRV) in immunoglobulin light-chain (AL) amyloidosis remains incompletely defined.

**Methods:**

In this retrospective cohort study, 56 patients with biopsy-proven AL amyloidosis and 33 age- and sex-comparable healthy controls underwent 24 h Holter monitoring. Time- and frequency-domain HRV parameters were recorded. Associations of HRV with cardiac involvement assessed by cardiac magnetic resonance (CMR), Mayo 2004 stage, and short-term treatment response were evaluated using group comparisons and multivariable regression.

**Results:**

Compared with controls, patients with AL amyloidosis had significantly higher mean heart rate and lower SDNN, total power (TP), and all frequency-domain components (ULF, VLF, LF, and HF) (all *P* < 0.05). Patients with CMR-defined cardiac involvement showed a higher prevalence of HRV abnormalities and lower SDNN, SDANN, pNN50, TP, ULF, VLF, and LF than CMR-negative patients (all nominal *P* < 0.05). In multivariable models, troponin remained independently associated with CMR-positive status, whereas HRV indices did not retain independent significance. By Mayo 2004 stage (I/II/III: 11/20/25), SDANN and VLF declined progressively with advancing stage (*P* < 0.05), and SDANN remained independently associated with higher stage after adjustment for troponin. In the exploratory subset with paired follow-up Holter recordings (*n* = 33), most HRV parameters did not change significantly after therapy; however, the coefficient of variation (CV) showed a statistically significant increase following treatment (*P* = 0.037).

**Conclusion:**

Reduced HRV is a prominent feature of AL amyloidosis and is independently associated with Mayo stage after adjustment for cardiac biomarkers. These findings position HRV as a non-invasive marker of autonomic involvement that complements established cardiac biomarkers in characterising disease severity in AL amyloidosis; whether this translates into independent prognostic value requires evaluation in prospective outcome studies.

## 1 Introduction

Amyloidosis is a group of rare, systemic, and progressive disorders, characterized by extracellular deposition of misfolded proteins, leading to multisystem organ dysfunction and substantial mortality (1, 2). Among various types of amyloidosis, transthyretin cardiac amyloidosis (ATTR), particularly the wild-type (ATTRwt), is now increasingly recognized as the most common form of cardiac amyloidosis (3), whereas systemic light chain amyloidosis (AL) remains a major systemic form with frequent cardiac involvement and high short-term mortality if untreated (4, 5). AL amyloidosis can affect multiple organ systems, including the heart, kidney, liver, nervous system, and gastrointestinal tract. Cardiac involvement represents the principal determinant of prognosis and is a leading cause of morbidity and mortality (6–8). Accordingly, accurate assessment of cardiac involvement and disease severity is central to risk stratification and clinical management (9–11).

Cardiac magnetic resonance (CMR) has become a key non-invasive modality for evaluating myocardial amyloid infiltration (12), while circulating cardiac biomarkers such as troponin and natriuretic peptides are routinely used for disease staging, prognostication, and monitoring treatment response (13, 14). Despite substantial advances in plasma cell–directed therapies that effectively suppress pathogenic light-chain production, a considerable proportion of patients continue to experience progressive organ dysfunction and adverse outcomes (11, 15, 16). Because current treatments primarily inhibit further amyloid formation rather than rapidly reversing existing tissue deposits, established organ damage often persists. Accordingly, accurate and comprehensive assessment of disease severity and multifaceted organ involvement—including autonomic nervous system dysfunction—is central to clinical management and risk stratification.

Heart rate variability (HRV) is a non-invasive physiological measure of cardiac autonomic modulation and has been widely studied in cardiovascular, neurological, and metabolic disorders (17, 18). While HRV reflects cardiac autonomic modulation, it is a functional index and is not a direct diagnostic test of autonomic neuropathy. Preliminary studies also indicate that patients with AL-amyloidosis may exhibit autonomic dysfunction, manifested by a significant decrease in HRV (19). However, existing studies have often been limited by their cross-sectional design, small sample size, and a lack of comprehensive integration with established cardiac biomarkers or validated disease staging systems. Consequently, the independent clinical significance of HRV in AL amyloidosis remains incompletely defined.

To address these gaps, we conducted a single-center retrospective cohort study to comprehensively assess autonomic function using 24 h HRV analysis in patients with biopsy-proven AL amyloidosis compared with healthy controls. We examined the relationship between HRV parameters and CMR-defined cardiac involvement, Mayo disease stage, and short-term treatment response. We hypothesized that reduced long-term HRV parameters would be associated with greater disease severity and provide information complementary to established cardiac biomarkers. By integrating HRV with imaging, biomarkers, and disease staging, this study aims to clarify the clinical relevance of autonomic dysfunction in AL amyloidosis and to explore whether HRV may serve as a complementary, non-invasive marker to support disease severity assessment.

## 2 Materials and methods

### 2.1 Study population and design

A total of 56 patients with newly diagnosed AL amyloidosis admitted to Henan Provincial People's Hospital between January 2018 and April 2025 were retrospectively enrolled. Amyloid diagnosis was established by tissue biopsy from involved organs and/or surrogate sites, including kidney, bone marrow, abdominal fat pad, gastrointestinal tract, and other organs, according to the clinical presentation and the recommendations of the Chinese Systemic Amyloidosis Collaborative Group. Congo red staining with characteristic apple-green birefringence under polarized light was required for histologic diagnosis of amyloid deposition. Subtyping as AL amyloidosis was based on immunohistochemical and/or immunofluorescence staining for immunoglobulin light chains in conjunction with hematologic and biochemical findings (serum and urine immunofixation electrophoresis and serum free light chain assay). All patients met the diagnostic criteria established by the Chinese Systemic Amyloidosis Collaborative Group. Patients were excluded if they had: (1) persistent atrial fibrillation, atrial flutter, or other sustained non-sinus rhythms, or frequent ectopy that precluded reliable HRV calculation based on the Holter report; (2) pacemaker dependency or implantable cardioverter-defibrillator (ICD) therapy with frequent pacing/therapy events that interfered with HRV assessment; (3) acute or hemodynamically unstable clinical conditions at the time of Holter monitoring; (4) documented severe autonomic dysfunction or neurologic disease expected to substantially confound HRV measures. For comparison, 33 healthy volunteers were recruited from the hospital physical examination center. Controls were age- and sex-comparable to the AL cohort and had no known cardiovascular disease. All controls had normal findings on physical examination, resting 12-lead electrocardiography, and transthoracic echocardiography. The study protocol complied with the Declaration of Helsinki and was approved by the Ethics Committee of Henan Provincial People's Hospital. Given the retrospective nature of the study, a waiver of written informed consent was granted where appropriate.

### 2.2 Clinical and laboratory measurements

Baseline demographic and clinical data, including age, sex, body mass index (BMI), comorbidities (hypertension and diabetes), and medication use were collected from all participants. Blood samples were obtained at baseline for the measurement of cardiac biomarkers. High-sensitivity cardiac troponin T (hs-cTnT) and N-terminal pro-B-type natriuretic peptide (NT-proBNP) levels in both AL amyloidosis patients and healthy controls were measured using Roche cobas h 232 platform (Roche Diagnostics, Mannheim, Germany).

### 2.3 HRV assessment

**Figure 1:**
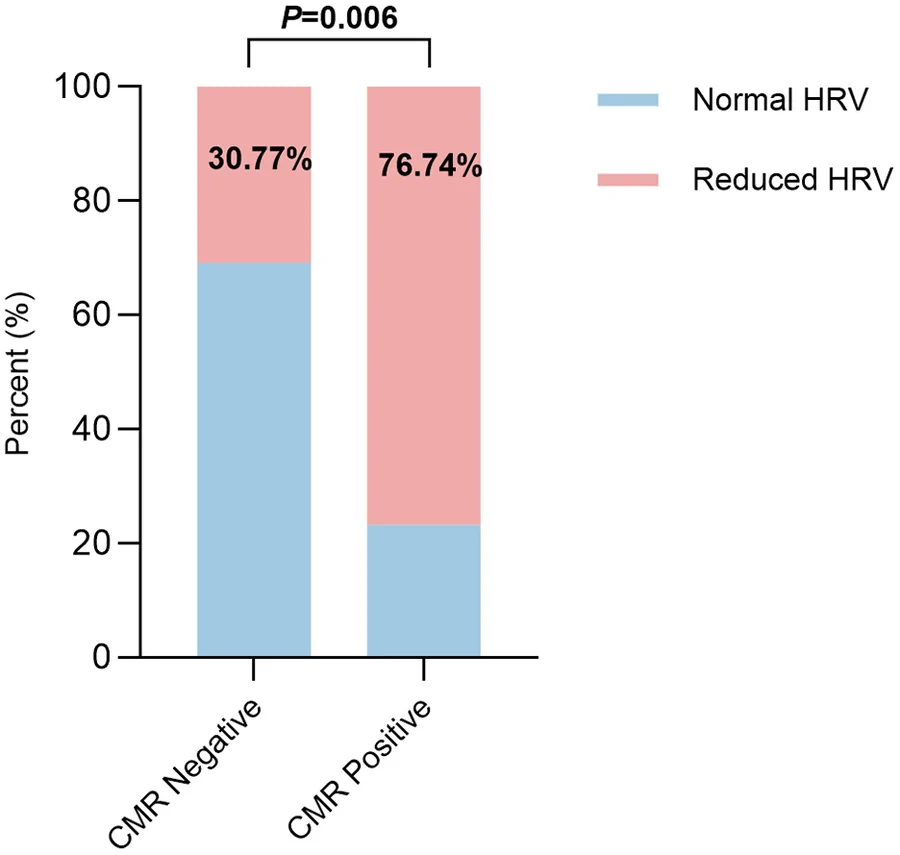
Prevalence oyyf reduced HRV (defined as SDNN ≤100 ms) in AL amyloidosis patients with CMR-negative and CMR-positive findings. Reduced HRV was present in 4 of 13 CMR-negative patients (30.77%) and in 33 of 43 CMR-positive patients (76.74%); the two groups were compared using the chi-square test with continuity correction. HRV, heart rate variability; SDNN, standard deviation of all normal-to-normal intervals; CMR, cardiac magnetic resonance.

All participants underwent 24 h ambulatory electrocardiographic monitoring using a TLC6000 dynamic ECG analysis system (Kangtai Medical Instrument Technology Co., China; sampling frequency 128 Hz). Recordings were analyzed by experienced technicians blinded to clinical data. HRV was calculated exclusively from normal-to-normal (NN) intervals. QRS complexes were detected and labeled by the software's automated algorithm using template matching and amplitude–timing criteria. Normal-to-normal (NN) intervals were then identified after exclusion of ectopic beats, artifacts, and noise segments based on RR interval outlier detection and signal quality indices. All automated annotations were rigorously verified and, where necessary, corrected by experienced technicians through beat-by-beat manual review. Only segments with adequate sinus rhythm and acceptable signal quality were retained for HRV analysis. Frequency-domain HRV parameters were computed from power spectral density estimates using fast Fourier transform (FFT) applied to successive 5-minute segments with standard windowing, and averaged over the 24-hour recording. To ensure standard comparability, HRV parameters were calculated and reported in accordance with the recommendations of the Task Force of the European Society of Cardiology and the North American Society of Pacing and Electrophysiology (1996) (27), which remain the most widely adopted reference for time- and frequency-domain HRV measurement and ensure comparability with prior studies. Time-domain indices, including the standard deviation of all NN intervals (SDNN), the root mean square of successive differences between adjacent NN intervals (rMSSD), the percentage of successive NN intervals differing by more than 50 ms (pNN50), and the coefficient of variation (CV), were calculated over the entire 24 h recording period. SDANN was specifically defined as the standard deviation of the average NN intervals calculated over successive standardized 5 min segments. For frequency-domain analysis, total power (TP), ultra-low-frequency (ULF), and very-low-frequency (VLF) were analyzed across the entire 24 h spectrum, whereas low-frequency (LF) and high-frequency (HF) power components were computed over successive 5-minute epochs and averaged over 24 h. All frequency parameters are expressed in ms^2^. “Reduced HRV” in this study was defined by a single literature-based criterion, SDNN ≤100 ms, which is the widely cited threshold for clinically relevant HRV impairment in the general cardiology literature (27, 29). This dichotomised definition was applied only to the prevalence comparison shown in Figure 1. All other HRV comparisons (across CMR status, Mayo 2004 stage, and pre- vs. post-treatment time points) were performed on continuous values, and no cohort-derived cutoffs were applied. While a small single-centre study reported that SDNN ≤50 ms predicts short-term mortality in AL amyloidosis (20), neither threshold has been externally validated in adequately powered prospective cohorts, and the categorisation used in the present study should therefore be interpreted accordingly.

### 2.4 Cardiac involvement assessment by CMR

In our center, CMR is routinely performed as part of the baseline evaluation for cardiac involvement in patients with biopsy-proven systemic AL amyloidosis, in accordance with institutional practice and contemporary guidelines. Accordingly, all 56 patients included in this study underwent CMR imaging. Both 24 h Holter monitoring and CMR were obtained at baseline, near the time of initial diagnosis, within a limited time window and before or shortly after initiation of systemic therapy, ensuring that both assessments reflected comparable stages of disease. Cardiac involvement was assessed using ECG-gated CMR performed on a 3.0-T scanner (Siemens Prisma, Siemens Healthineers, Germany) according to routine clinical protocols. The diagnostic criteria for positive CMR included sequences acquired from four-chamber, two-chamber, and three-chamber apical views, as well as short-axis views at the basal, mid-ventricular, and apical levels of the left ventricle. Late gadolinium enhancement (LGE) images were obtained 8–10 min after intravenous bolus administration of a gadolinium-based contrast agent. The presence of typical diffuse subendocardial or transmural LGE with abnormal gadolinium kinetics was determined by two experienced radiologists. Patients without these findings were classified as CMR negative. Cardiac involvement was thus defined by LGE alone; parametric mapping (native T1 or extracellular volume) was not part of the routine protocol during the study period, so early infiltration without LGE would not have been detected.

### 2.5 Assessment of treatment effects

According to the Mayo 2004 staging system, AL patients were staged at baseline. All patients received guideline-recommended regimens based on proteasome inhibitors (bortezomib, carfilzomib, or ixazomib), immunomodulators (lenalidomide or pomalidomide), cyclophosphamide, and/or daratumumab. Treatment response was evaluated after a minimum of 3 months of therapy. Follow-up evaluations generally included clinical assessment and laboratory testing. Repeat HRV analysis was performed in a subset of patients who underwent follow-up 24-hour Holter monitoring. Complete response was defined as negative serum and urine immunofixation electrophoresis with normalization of serum free light chain level and ratio.

Of the 56 enrolled patients, 33 (58.9%) had paired baseline and follow-up 24 h Holter recordings available for treatment-response analysis. Follow-up Holter monitoring was not mandated by a prespecified protocol but was performed at the discretion of treating physicians as part of routine clinical care, typically in patients who returned for scheduled appointments and consented to repeat monitoring. To characterize potential selection bias, baseline clinical characteristics were compared between patients with and without follow-up Holter recordings.

### 2.6 Statistical analysis

Continuous variables were reported as mean ± standard deviation for normally distributed data or median (interquartile range) for non-normally distributed data. Normality was assessed using the Shapiro–Wilk test. Categorical variables were reported as counts and percentages. Comparisons between two independent groups were performed using the independent-sample *t*-test or the Mann–Whitney U test, as appropriate. Categorical variables were compared using the Chi-square test or Fisher's exact test, as appropriate. For comparisons across multiple groups, analysis of variance (ANOVA) or the Kruskal–Wallis H test was used. Paired comparisons for pre- and post-treatment HRV parameters used paired t-test or Wilcoxon signed-rank test. Given the exploratory nature of the HRV analyses and the limited sample size, no formal adjustment for multiple comparisons was applied; therefore, *P* values should be interpreted as nominal. Biomarker variables with skewed distributions were log-transformed prior to the logistic regression analyses of CMR positivity; in the ordinal models of Mayo stage, hs-cTnT was analysed on its original scale. To identify factors associated with CMR-positive cardiac involvement, univariable logistic regression analysis was conducted, and variables with a univariable *P*-value < 0.05 were entered into a multivariable logistic regression model. To address the risk of overfitting in the CMR binary logistic model, backward stepwise selection was applied given the limited number of events in the minority class; the events-per-variable (EPV) criterion and final model specification are reported in Supplementary Table S2. Collinearity among candidate predictors was assessed using variance inflation factors (VIF) before model reduction. NT-proBNP and hs-cTnT are strongly correlated in AL amyloidosis; both were therefore offered to the selection procedure, and VIF values were inspected to confirm that the reduced model was not distorted by their correlation. As a sensitivity analysis, L1-penalized least absolute shrinkage and selection operator (LASSO) logistic regression with the penalty parameter selected by ten-fold cross-validation was additionally applied. The initial choice of ln hs-cTnT and SDANN as primary predictors for the CMR and ordinal logistic models, respectively, was guided by *a priori* clinical hypotheses rather than data-driven variable selection; these predictors were pre-specified based on their established pathophysiological roles in AL amyloidosis. To assess factors associated with advancing amyloidosis disease stage, univariable and multivariable ordinal logistic regression analyses were conducted. The proportional-odds assumption for the ordinal logistic regression model was examined by comparing cumulative-logit coefficients across stage cutpoints and formally tested using the test of parallel lines. Missing data were addressed using a complete case analysis approach. The extent of missingness was limited. Demographic, clinical, biomarker, and time-domain HRV variables (age, sex, hs-cTnT, NT-proBNP, mean heart rate, SDNN, SDANN, rMSSD, pNN50) were complete for all 56 patients (0% missing). The coefficient of variation and the five frequency-domain indices (TP, ULF, VLF, LF, and HF) were unavailable in the same 2 of 56 patients (3.6%) and in 2 of 33 controls (6.1%), in whom the Holter system did not output these report fields; missingness did not differ across CMR or Mayo-stage groups. The final complete-case sample was therefore 56 for all baseline analyses except those involving the coefficient of variation or frequency-domain indices (*n* = 54); the numbers of paired observations available for each parameter in the treatment-response analysis are specified in the footnote to Table 1, where the same report-output limitation affected a larger fraction of the repeat recordings (coefficient of variation available in 27 of 33 pairs, frequency-domain indices in 29 of 33). All statistical analyses were conducted using SPSS Statistics version 26.0 and R statistical software version 4.2.0. A *P*-value of <0.05 was considered statistically significant.

**Table 1:** HRV in patients with AL amyloidosis before and after treatment.

Variables	Before treatment(*n* = 33)	After treatment(*n* = 33)	*P*
Follow-up duration (months) median (IQR)	–	9.50 (3.00-14.75)	–
Mean heart rate (bpm)	74.64 ± 8.03	76.18 ± 12.42	0.519
SDNN (ms)	101.48 ± 29.61	99.01 ± 36.27	0.679
SDANN (ms)	114.71 ± 39.58	126.18 ± 74.65	0.860
rMSSD (ms)	32.80 (22.70-67.20)	35.20 (21.60-50.50)	0.971
pNN50 (%)	3.90 (1.35–11.50) a	2.75 (1.20–6.15) a	0.395
CV	0.045 ± 0.018 b	0.062 ± 0.033 b	0.037
TP (ms^2^)	4967.00 (2,608.25–7,480.75) c	3,413.80 (1,662.00–6,854.05) c	0.121
ULF (ms^2^)	2,361.80 (1,799.30–5,402.20) c	1,911.40 (956.00–3,988.20) c	0.072
VLF (ms^2^)	826.80 (445.40–1,246.45) c	873.30 (416.90–1,826.70) c	0.262
LF (ms^2^)	138.50 (45.90–316.25) c	144.10 (89.50–228.55) c	0.766
HF (ms^2^)	95.80 (32.85–404.95) c	102.00 (41.45–177.20) c	0.670

HRV, heart rate variability; AL, light chain amyloidosis; IQR, interquartile range; SDNN, standard deviation of all normal-to-normal RR intervals; SDANN, standard deviation of the average normal-to-normal RR intervals for all 5 min segments; rMSSD, root mean square of successive differences between adjacent normal RR intervals; pNN50, percentage of successive normal RR intervals differing by more than 50 ms; CV, coefficient of variation; TP, total power; ULF, ultra-low frequency; VLF, very-low frequency; LF, low frequency; HF, high frequency. Data are presented as mean ± SD for approximately symmetrically distributed variables and as median (interquartile range) for skewed variables, consistent with the Task Force (1,996) recommendation that frequency-domain powers be summarised by robust measures. Paired comparisons were performed using the paired t-test when the distribution of within-patient differences was normal and the Wilcoxon signed-rank test otherwise. All *P* values are nominal and were not corrected for multiple comparisons; after Bonferroni correction for the 11 parameters compared (*α* = 0.0045), no parameter reached significance. Numbers of available paired observations: a (*n* = 32); b (*n* = 27); c (*n* = 29); all other parameters *n* = 33.

## 3 Results

### 3.1 Baseline characteristics and HRV impairment in AL amyloidosis

A total of 56 patients with AL amyloidosis and 33 healthy controls were included. The two groups were comparable in age (62.68 ± 9.13 vs. 65.86 ± 11.32 years, *P* = 0.152) and sex distribution (male/female: 29/27 vs. 22/11, *P* = 0.170) (Table 2). BMI and smoking status were also similar between groups. In contrast, patients with AL amyloidosis had a higher prevalence of diabetes mellitus (DM) and hypertension, as well as more frequent use of angiotensin-converting enzyme inhibitors/angiotensin receptor blockers (ACEI/ARB) and β-blockers. Compared with controls, the AL group showed significantly higher median levels of hs-cTnT (*P* = 0.001) and NT-proBNP (*P* < 0.001). Mean heart rate was significantly higher in the AL group than in controls (77.57 ± 10.50 bpm vs. 71.61 ± 11.98 bpm, *P* = 0.016). Time-domain HRV indices were significantly lower in AL patients, including SDNN (91.76 ± 35.11 vs. 125.83 ± 26.36 ms, *P* < 0.001) and CV (0.045 ± 0.028 vs. 0.067 ± 0.028, *P* = 0.001). Besides, pNN50 was significantly lower in AL patients (*P* = 0.038), whereas the reduction in rMSSD did not reach statistical significance (*P* = 0.065). In the frequency domain, all five indices (TP, ULF, VLF, LF, and HF) were significantly lower in AL patients than in controls. Given that β-blockers and ACEI/ARB may influence heart rate and HRV, we assessed their potential confounding effects. Among AL patients, 14 (25.00%) received β-blockers and 16 (28.57%) received ACEI/ARB. HRV parameters did not differ significantly between AL patients treated with ACEI/ARB and those not treated with ACEI/ARB. In contrast, β-blocker use was associated with a lower mean heart rate (68.64 ± 5.67 vs. 80.21 ± 10.12 bpm, *P* < 0.001) and a higher SDNN (111.19 ± 41.31 vs. 86.11 ± 30.74 ms, *P* = 0.019) (Supplementary Table S1). Importantly, after adjusting for β-blocker use, the between-group comparisons remained materially unchanged: HRV indices in the AL group were still significantly lower than those in healthy controls (all *P* < 0.05).

**Table 2:** characteristics and HRV between AL amyloidosis and control groups.

Variables	Control(*n* = 33)	AL Amyloidosis(*n* = 56)	*P*
Age (years)	65.86 ± 11.32	62.68 ± 9.13	0.152
Sex (male/female)	22/11	29/27	0.170
Body mass index (kg/m^2^)	23.88 ± 3.68	23.23 ± 2.98	0.409
Smoke (*n*, %)	9 (27.27%)	20 (35.71%)	0.412
Diabetic mellitus (*n*, %)	0 (0.0)	8 (14.29%)	0.023
Hypertension (*n*, %)	0 (0.0)	23 (41.07%)	<0.001
Mayo 2004 stage (I/II/III, *n*)	–	11/20/25	–
ACEI/ARB (*n*, %)	0 (0.0)	16 (28.57%)	0.001
β-Receptor blocker (*n*, %)	0 (0.0)	14 (25.00%)	0.002
NT-pro BNP (pg/mL)	118.00 (92.50–200.25)	2,785.00 (478.00–5,002.50)	<0.001
hs-cTnT (ng/L)	3.40 (1.92–12.37)	22.84 (3.84–45.25)	0.001
Mean heart rate (bpm)	71.61 ± 11.98	77.57 ± 10.50	0.016
SDNN (ms)	125.83 ± 26.36	91.76 ± 35.11	<0.001
SDANN (ms)	127.36 ± 45.20	113.53 ± 45.12	0.166
rMSSD (ms)	38.00 (26.25–57.50)	28.40 (18.63–57.28)	0.065
pNN50 (%)	5.70 (1.75–14.75)	2.40 (0.83–5.30)	0.038
CV	0.067 ± 0.028	0.045 ± 0.028	0.001
TP (ms^2^)	6,739.80 (5,472.90–8,818.80)	4,631.30 (2,342.58–5,768.93)	<0.001
ULF (ms^2^)	4,869.80 (2,776.50–6,960.60)	2,400.60 (1,302.33–4,571.05)	<0.001
VLF (ms^2^)	1,613.80 (883.00–2,147.60)	737.50 (303.75–1,056.13)	<0.001
LF (ms^2^)	209.20 (129.40–447.50)	65.40 (35.98–201.50)	<0.001
HF (ms^2^)	157.60 (80.00–384.40)	67.05 (26.53–209.15)	0.008

HRV, heart rate variability; AL, light chain amyloidosis; ACEI, angiotensin-converting enzyme inhibitors; ARB, angiotensin receptor blockers; NT-proBNP, N-terminal pro-B-type natriuretic peptide; hs-cTnT, high-sensitivity cardiac troponin T; SDNN, standard deviation of all normal-to-normal RR intervals; SDANN, standard deviation of the average normal-to-normal RR intervals for all 5 min segments; rMSSD, root mean square of successive differences between adjacent normal RR intervals; pNN50, percentage of successive normal RR intervals differing by more than 50 ms; CV, coefficient of variation; TP, total power; ULF, ultra-low frequency; VLF, very-low frequency; LF, low frequency; HF, high frequency. The coefficient of variation and the frequency-domain indices (TP, ULF, VLF, LF, HF) were available in 31 of 33 controls and 54 of 56 patients; all other variables were available in all participants. All *P* values are nominal and were not corrected for multiple comparisons.

### 3.2 HRV and CMR-defined cardiac involvement

Among the 56 AL amyloidosis (Table 3), 13 patients (23.2%) were classified as CMR-negative and 43 (76.8%) as CMR-positive. Baseline age (62.38 ± 8.07 vs. 62.77 ± 9.52 years, *P* = 0.896) and mean heart rate (76.54 ± 8.22 vs. 77.88 ± 11.16 bpm, *P* = 0.689) did not differ significantly between the two groups. Patients with CMR-positive findings had significantly higher median NT-proBNP levels [3,260.00 (2,040.00–5,550.00) pg/mL vs. 288.00 (89.50–550.00) pg/mL, *P* < 0.001] and higher median hs-cTnT levels [28.40 (15.10–72.77) ng/L vs. 1.70 (1.50–4.16) ng/L, *P* < 0.001] compared to those with CMR-negative findings. The prevalence of reduced HRV was markedly higher in the CMR-positive group than in the CMR-negative group (76.74% vs. 30.77%, *P* = 0.006) (Figure 1), indicating that HRV abnormalities were more common among patients with imaging evidence of cardiac involvement. Compared with the CMR-negative group, patients with CMR-positive findings exhibited lower time-domain indices (SDNN, SDANN, and pNN50) and lower frequency-domain indices (TP, ULF, VLF, and LF), all of which reached nominal significance (all *P* < 0.05, uncorrected for multiple comparisons). The reduction in HF was of borderline significance (*P* = 0.050), whereas rMSSD did not differ significantly between the two groups. To identify factors independently associated with CMR-defined cardiac involvement, we performed univariable logistic regression analysis. Elevated levels of log-transformed NT-proBNP and hs-cTnT, lower SDANN and ULF, and male sex were significantly associated with CMR-positive cardiac involvement. However, after stepwise selection in the multivariable model, only log-transformed hs-cTnT remained independently associated with CMR-positive cardiac involvement (OR 7.19, 95% CI 2.48–20.84, *P* < 0.001) (Table 4). Of the five candidate variables meeting the univariable threshold (log NT-proBNP, log hs-cTnT, SDANN, ULF, and sex), simultaneous entry would have yielded approximately 2.6 events per variable—below the conventional threshold of 10; backward stepwise selection was therefore applied, and the final model retained a single predictor (log hs-cTnT), corresponding to 13 events per variable. VIF values for all candidate predictors were uniformly low (all <3.0). In the LASSO sensitivity analysis, four variables retained non-zero coefficients (log hs-cTnT, log NT-proBNP, ULF, and sex), among which log hs-cTnT carried the largest coefficient. The two approaches therefore agree in identifying log hs-cTnT as the strongest predictor, while the retention of additional variables under penalization indicates that a larger sample might support a less parsimonious model than the one adopted here. In LASSO-penalized logistic regression of CMR positivity, log-transformed hs-cTnT carried the largest non-zero coefficient after penalization, in agreement with the single predictor retained by the conventional stepwise model (Supplementary Table S2). To assess the potential confounding effects of beta-blocker use and cardiovascular comorbidities on the association between ln hs-cTnT and CMR positivity, we entered each potential confounder individually into the base logistic model (owing to the EPV constraint of 13 CMR-negative events). The association of ln hs-cTnT with CMR positivity remained robust across all confounding models (adjusted OR 6.83–9.76, all *P* ≤ 0.002). Beta-blocker use (OR = 0.48, 95% CI 0.03–7.21, *P* = 0.59), diabetes (*P* = 0.47), hypertension (*P* = 0.31), mean heart rate (*P* = 0.32), and ACEI/ARB use (*P* = 0.78) were not independently associated with CMR positivity. Beta-blocker use was infrequent among CMR-negative patients (2 of 13 vs. 12 of 43 CMR-positive patients), which accounts for the wide confidence interval of that covariate and limits the precision with which it can be estimated in this model. In a sensitivity analysis restricted to non-beta-blocker users (*n* = 42), the association persisted (OR = 6.27, 95% CI 1.95–20.18, *P* = 0.002), confirming that the observed association between ln hs-cTnT and CMR involvement is not attributable to confounding by medication use or common cardiovascular comorbidities.

**Table 3:** HRV in CMR-negative vs. CMR-positive AL amyloidosis.

Variables	CMR-negative(*n* = 13)	CMR-positive(*n* = 43)	*P*
Age (years)	62.38 ± 8.07	62.77 ± 9.52	0.896
Sex (male/female)	3/10	26/17	0.018
NT-proBNP (pg/mL)	288.00 (89.50–550.00)	3,260.00 (2,040.00–5,550.00)	<0.001
hs-cTnT (ng/L)	1.70 (1.50–4.16)	28.40 (15.10–72.77)	<0.001
Mean heart rate (bpm)	76.54 ± 8.22	77.88 ± 11.16	0.689
SDNN (ms)	108.68 ± 22.82	86.64 ± 36.74	0.046
SDANN (ms)	137.06 ± 34.75	106.41 ± 45.80	0.031
rMSSD (ms)	30.60 (24.50–55.95)	25.70 (17.80–60.50)	0.165
pNN50 (%)	4.20 (2.75–8.90)	1.40 (0.70–3.90)	0.010
CV	0.051 ± 0.022	0.043 ± 0.030	0.385
TP (ms^2^)	5,775.00 (3,988.05–8,818.35)	3,946.30 (2,137.75–5,419.20)	0.020
ULF (ms^2^)	4,415.00 (2,061.70–6,601.50)	2,037.36 (1,202.20–4,276.90)	0.034
VLF (ms^2^)	893.00 (773.70–1,275.50)	501.20 (258.90–934.60)	0.018
LF (ms^2^)	137.90 (72.55–301.80)	50.40 (27.50–204.80)	0.043
HF (ms^2^)	80.10 (67.55–306.25)	52.90 (21.45–217.05)	0.050

HRV, heart rate variability; CMR, cardiac magnetic resonance; AL, light chain amyloidosis; NT-proBNP, N-terminal pro-B-type natriuretic peptide; hs-cTnT, high-sensitivity cardiac troponin T; SDNN, standard deviation of all normal-to-normal RR intervals; SDANN, standard deviation of the average normal-to-normal RR intervals for all 5 min segments; rMSSD, root mean square of successive differences between adjacent normal RR intervals; pNN50, percentage of successive normal RR intervals differing by more than 50 ms; CV, coefficient of variation; TP, total power; ULF, ultra-low frequency; VLF, very-low frequency; LF, low frequency; HF, high frequency. The coefficient of variation and the frequency-domain indices (TP, ULF, VLF, LF, HF) were available in 13 CMR-negative and 41 CMR-positive patients; all other variables were available in all 56 patients. All *P* values are nominal and were not corrected for multiple comparisons.

**Table 4:** Predictive value of HRV and cardiac biomarkers for CMR-defined cardiac involvement.

Variables	Univariable					Multivariable				
	β	S.E	Z	*P*	OR (95%CI)	β	S.E	Z	*P*	OR (95%CI)
Log NT-proBNP	1.20	0.34	3.55	<0.01	3.33 (1.71–6.48)					
Log hs-cTnT	1.97	0.54	3.63	<0.001	7.19 (2.48–20.84)	1.97	0.54	3.63	<0.001	7.19 (2.48–20.84)
SDANN (ms)	−0.015	0.007	−2.043	0.041	0.985 (0.971–0.999)					
pNN50 (%)	−0.00	0.03	−0.02	0.98	1.00 (0.95–1.05)					
TP (ms²)	−0.00	0.00	−1.33	0.18	1.00 (1.00–1.00)					
ULF (ms²)	−0.01	0.00	−2.14	0.03	0.99 (0.99–0.99)					
VLF (ms²)	−0.00	0.00	−0.23	0.82	1.00 (1.00–1.00)					
LF (ms²)	0.00	0.00	0.44	0.66	1.00 (1.00–1.00)					
SEX										
Famale					1.00 (Reference)					
Male	1.63	0.73	2.24	0.03	5.10 (1.22–21.25)					

HRV, heart rate variability; CMR, cardiac magnetic resonance; NT-proBNP, N-terminal pro-B-type natriuretic peptide; hs-cTnT, high-sensitivity cardiac troponin T; SDANN, standard deviation of the average normal-to-normal RR intervals for all 5-minute segments; pNN50, percentage of successive normal RR intervals differing by more than 50 ms; TP, total power; ULF, ultra-low frequency; VLF, very-low frequency; LF, low frequency; OR, odds ratio; CI, confidence interval.

### 3.3 HRV across disease stages

**Figure 2:**
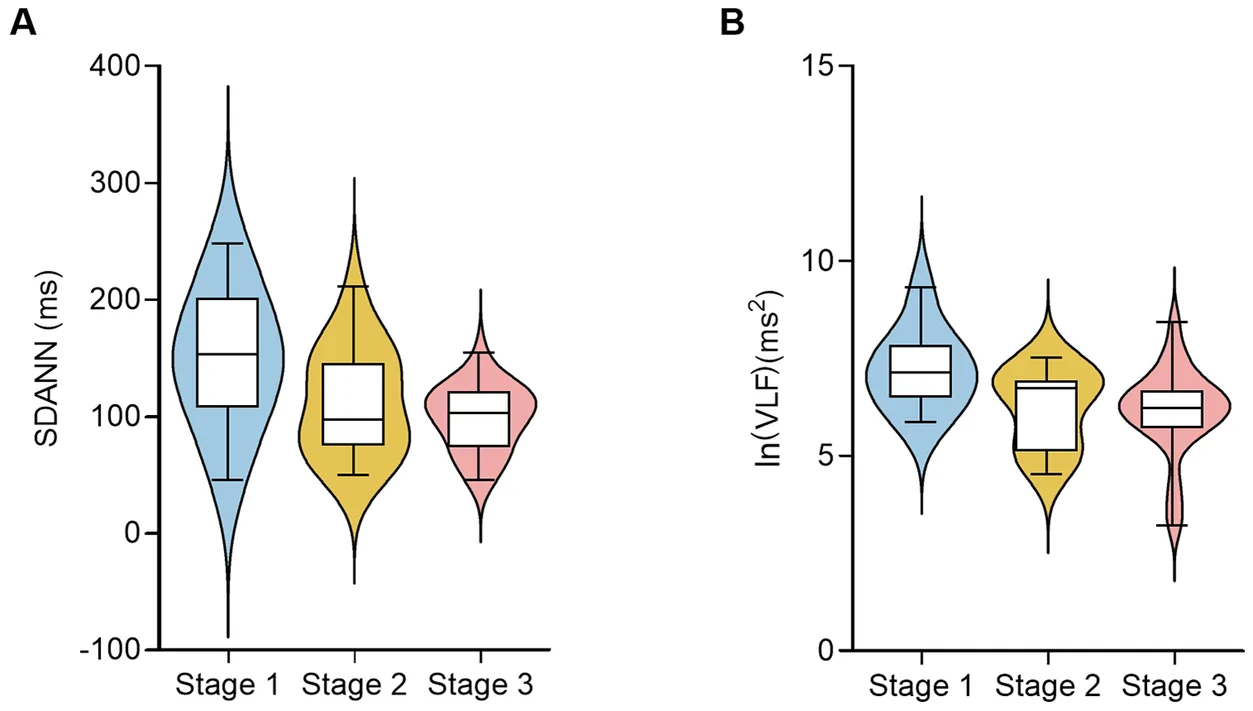
Trends in SDANN **(A)** and VLF **(B)** across mayo 2004 disease stages in patients with AL amyloidosis. Group sizes were 11, 20, and 25 for stages I, II, and III for SDANN, and 10, 19, and 25 for VLF. Violin plots show the kernel density of each distribution; the superimposed box marks the median and interquartile range, and the whiskers span the minimum and maximum observed values. Values in panel B are plotted on the natural-logarithmic scale; untransformed VLF values are reported in Table 5. Differences across stages were significant for both SDANN (*P* = 0.006) and VLF (*P* = 0.045). SDANN, standard deviation of the average normal-to-normal RR intervals for all 5 min segments; VLF, very-low frequency.

Patients were categorized according to the Mayo 2004 staging system into stage I (*n* = 11), stage II (*n* = 20), and stage III (*n* = 25). Age, sex distribution, and mean heart rate did not differ significantly across the stages (all *P* > 0.05) (Table 5). In contrast, cardiac biomarkers increased progressively with advancing stage, with NT-proBNP and hs-cTnT showing significant stepwise elevations. Analysis of HRV parameters revealed a graded decline in HRV indices with increasing disease severity. SDANN decreased significantly across Mayo stages, with the highest values in stage I and the lowest in stage III (*P* = 0.006). VLF power also demonstrated a significant downward trend with advancing stage (*P* = 0.045) (Figure 2). Other HRV parameters, including SDNN, rMSSD, and HF power, did not differ significantly among the three stages. To further investigate factors independently associated with advanced amyloidosis disease stages, an ordinal logistic regression analysis was performed (Table 6). In univariable models, reduced SDANN and elevated hs-cTnT levels were significantly associated with higher Mayo stage. In multivariable model, after adjustment and stepwise selection, SDANN and hs-cTnT remained independently associated with advancing disease stages. Specifically, for every unit decrease in SDANN, the odds of progressing to a higher disease stage increased by 1.5% (OR = 0.985, 95% CI: 0.972–0.998, *P* = 0.021), whereas higher hs-cTnT was independently associated with increased odds of higher stage (OR = 1.044, 95% CI: 1.015–1.075, *P* = 0.003). The proportional-odds assumption for the ordinal logistic model was confirmed using the test of parallel lines (*P* = 0.571), supporting the validity of the proportional odds specification. To address potential confounding, individual covariates were explored in the Mayo ordinal logistic model. The associations of SDANN (OR = 0.985, 95% CI 0.972–0.998, *P* = 0.024) and hs-cTnT (OR = 1.045, 95% CI 1.015–1.075, *P* = 0.003) with Mayo stage remained stable after adjustment for beta-blocker use. Neither beta-blocker use (*P* = 0.63), diabetes (*P* = 0.17), nor hypertension (*P* = 0.07) was independently associated with disease stage. When mean heart rate was additionally included, the SDANN association attenuated (*P* = 0.092), reflecting collinearity between heart rate and SDANN in beta-blocker users rather than a confounding effect. In the sensitivity analysis excluding all 14 beta-blocker users (*n* = 42), hs-cTnT remained significantly associated with Mayo stage (OR = 1.048, 95% CI 1.010–1.087, *P* = 0.012), and the SDANN association was borderline (*P* = 0.053), consistent with reduced statistical power in the smaller sample.

**Table 5:** Comparison of HRV across mayo stage groups in AL amyloidosis.

Characteristics	Stage I (*n* = 11)	Stage II (*n* = 20)	Stage III (*n* = 25)	*P*
Age (years)	64.82 ± 8.78	62.55 ± 10.66	61.84 ± 8.14	0.672
Sex(male/female)	4/7	11/9	14/11	0.520
NT-proBNP (pg/mL)	337.00 (115.00–2,340.00)	3,104.00 (481.00–5,135.00)	3,260.00 (1,463.00–7,160.00)	0.007
hs-cTnT (ng/L)	3.60 (1.50–21.00)	14.51 (5.03–32.50)	40.60 (23.95–107.45)	0.001
Mean heart rate (bpm)	71.36 ± 5.92	78.05 ± 11.37	79.36 ± 10.63	0.098
SDNN (ms)	111.31 ± 44.64	90.48 ± 35.13	84.18 ± 27.95	0.099
SDANN (ms)	148.99 ± 58.87	112.16 ± 45.69	99.02 ± 27.77	0.007
rMSSD (ms)	42.00 (22.30–64.30)	26.05 (19.05–34.05)	28.60 (16.30–64.05)	0.424
pNN50 (%)	4.20 (2.50–18.10)	2.40 (0.83–4.95)	2.10 (0.70–4.25)	0.147
CV	0.060 ± 0.040	0.040 ± 0.014	0.042 ± 0.029	0.160
TP (ms^2^)	6,401.30 (3,469.83–11,666.70)	4,682.90 (2,119.90–5,766.90)	3,601.90 (2,330.35–5,419.20)	0.106
ULF (ms^2^)	2,519.15 (1,653.90–7,693.38)	3,414.80 (1,535.30–5,134.40)	2,037.36 (1,123.80–4,434.45)	0.411
VLF (ms^2^)	1,246.45 (683.83–2,562.23)	848.60 (165.40–1,034.80)	501.20 (301.80–800.45)	0.034
LF (ms^2^)	197.65 (69.33–587.48)	51.20 (20.70–157.30)	50.60 (35.85–193.20)	0.053
HF (ms^2^)	152.40 (69.28–533.15)	54.10 (28.70–112.20)	62.10(20.40–296.60)	0.116

HRV, heart rate variability; AL, light chain amyloidosis; NT-proBNP, N-terminal pro-B-type natriuretic peptide; hs-cTnT, high-sensitivity cardiac troponin T; SDNN, standard deviation of all normal-to-normal RR intervals; SDANN, standard deviation of the average normal-to-normal RR intervals for all 5 min segments; rMSSD, root mean square of successive differences between adjacent normal RR intervals; pNN50, percentage of successive normal RR intervals differing by more than 50 ms; CV, coefficient of variation; TP, total power; ULF, ultra-low frequency; VLF, very-low frequency; LF, low frequency; HF, high frequency. The coefficient of variation and the frequency-domain indices (TP, ULF, VLF, LF, HF) were available in 10 of 11 stage I, 19 of 20 stage II, and 25 of 25 stage III patients; all other variables were available in all 56 patients. All *P* values are nominal and were not corrected for multiple comparisons.

**Table 6:** Factors associated with advanced mayo disease stage in AL amyloidosis.

Variables	Univariable					Multivariable				
	β	S.E	Z	*P*	OR (95%CI)	β	S.E	Z	*P*	OR (95%CI)
SDANN (ms)	−0.017	0.006	−2.822	0.005	0.983 (0.971–0.995)	−0.016	0.007	−2.308	0.021	0.985 (0.972–0.998)
VLF (ms^2^)	−0.00	0.00	−1.80	0.07	1.00 (1.00–1.00)					
NT-proBNP (pg/mL)	0.00	0.00	1.61	0.11	1.00 (1.00–1.00)					
hs-cTnT (ng/L)	0.049	0.015	3.232	0.001	1.050 (1.019–1.081)	0.043	0.015	2.961	0.003	1.044 (1.015–1.075)

AL, light chain amyloidosis; SDANN, standard deviation of the average normal-to-normal RR intervals for all 5 min segments; VLF, very-low frequency; NT-proBNP, N-terminal pro-B-type natriuretic peptide; hs-cTnT, high-sensitivity cardiac troponin T; OR, odds ratio; CI, confidence interval. hs-cTnT was analysed on its original scale in this model, so the odds ratio corresponds to a 1 ng/L increment; the log-transformed form was used only in the logistic models of CMR positivity (Table 4). β, regression coefficient; S.E., standard error; Z, Wald statistic.

### 3.4 Effect of treatment on HRV

Among the 56 patients, 42 (75%) achieved complete hematologic remission after treatment. Paired baseline and follow-up Holter recordings were available in 33 patients, and the median interval from treatment initiation to repeat monitoring was 9.50 months (interquartile range 3.00–14.75 months). Compared with baseline, no significant changes were observed in mean heart rate, in the time-domain HRV indices other than the coefficient of variation, or in the frequency-domain measures at follow-up (Table 1). Among the individual HRV parameters, the coefficient of variation (CV) showed a statistically significant increase after treatment [before: 0.045 ± 0.018 (*n* = 27) vs. after: 0.062 ± 0.033, *P* = 0.037]. All other HRV parameters did not change significantly (all *P* ≥ 0.07), and this single CV finding should be interpreted with caution given the multiple comparisons performed and the exploratory nature of the analysis.

To assess potential selection bias, baseline characteristics were compared between the 33 patients with paired Holter recordings and the 23 without follow-up recordings (Supplementary Table S3). The two groups did not differ significantly in age, sex, Mayo stage, or hs-cTnT (all *P* > 0.05); however, NT-proBNP was lower in the follow-up group [2,408.94 ± 2,591.10 vs. 5,882.13 ± 6,724.77 pg/mL, *P* = 0.024] and baseline SDNN was higher [101.48 ± 29.61 vs. 77.81 ± 38.23 ms, *P* = 0.004], suggesting that patients who completed repeat monitoring may represent a somewhat more clinically stable or adherent subset. This treatment-response analysis should be regarded as exploratory only. Repeat Holter monitoring was available in a non-random subset (*n* = 33), and the heterogeneity of treatment regimens and variable follow-up duration further constrain interpretation; survivorship and selection bias cannot be excluded (see Discussion).

## 4 Discussion

In this study, we comprehensively evaluated autonomic function in patients with AL amyloidosis using 24 h HRV analysis and identified several clinically meaningful findings. First, patients with AL exhibited a marked and generalized reduction in HRV compared with healthy controls, indicating significant impairment of autonomic regulation. Second, HRV indices were significantly lower in patients with CMR-defined cardiac involvement, underscoring the close link between autonomic dysfunction and myocardial amyloid infiltration. Third, and most importantly, reduced long-term HRV—represented by SDANN, was independently associated with advanced disease stage, suggesting that HRV may provide complementary information for disease severity assessment.

Our findings confirm and extend previous observations of autonomic dysfunction in amyloidosis. Studies have reported reduced HRV in patients with cardiac amyloidosis, supporting the presence of autonomic impairment in this population (19, 20). However, prior studies were limited by small sample sizes, heterogeneous amyloid subtypes, or a primary focus on cardiac involvement without systematic integration of disease staging or multivariable adjustment for cardiac biomarkers. By restricting our cohort to biopsy-proven AL amyloidosis and integrating HRV analysis with CMR imaging, circulating biomarkers, and Mayo staging, our study provides a more comprehensive evaluation of the clinical relevance of autonomic dysfunction in this specific disease context. Our findings also align with a growing body of work on electrical and autonomic markers in systemic amyloidosis assessed by ECG and ambulatory monitoring. Prior Holter-based studies have documented severe reductions in time-domain HRV—particularly in SDNN and SDANN—as a hallmark of AL cardiac amyloidosis, distinguishing it from other causes of left ventricular hypertrophy with high discriminative accuracy (SDNN AUC 0.865, SDANN AUC 0.834) (19). More recently, the central role of vagal withdrawal and autonomic dysregulation has been comprehensively characterised as a distinct pathophysiological feature of cardiac amyloidosis, irrespective of subtype (26). On the electrocardiographic side, in a multicentre cohort of 356 patients spanning AL, hereditary, and wild-type transthyretin amyloidosis, QRS duration at baseline remained independently associated with survival after multivariable adjustment (HR 1.78, 95% CI 1.13–2.8) (28). Collectively, these observations situate the present findings within an emerging landscape in which both depolarization/repolarization abnormalities (ECG) and autonomic modulation (Holter-derived HRV) contribute non-redundant, complementary information on disease severity in systemic amyloidosis.

CMR, as a multiparametric and non-invasive imaging modality, has become a key tool for the evaluation of cardiac amyloidosis, providing high sensitivity and specificity for detecting myocardial involvement and quantifying its extent and severity (21–24). In our cohort, patients were stratified according to CMR findings, and a markedly higher proportion of those with CMR-positive cardiac involvement exhibited reduced HRV compared with CMR-negative patients (76.7% vs. 30.8%). This indicates that HRV abnormalities are more prevalent among patients with imaging evidence of myocardial amyloid infiltration, while the presence of reduced HRV in a subset of CMR-negative patients suggests that HRV analysis may detect autonomic dysfunction even in the absence of overt structural abnormalities on CMR and thus may provide adjunctive physiological information. Furthermore, time-domain indices (SDANN and pNN50) and frequency-domain measures (TP, ULF, and VLF) were significantly lower in the CMR-positive group, and HRV parameters declined progressively with higher Mayo disease stage, supporting a close association between the severity of autonomic dysfunction and the burden of cardiac amyloid involvement. These findings align with the concept that more extensive myocardial infiltration and functional impairment are accompanied by more pronounced reductions in HRV. Whether this translates into a higher risk of adverse outcomes was not assessed here; late gadolinium enhancement itself carries established prognostic weight in cardiac amyloidosis (25), but the present study recorded no clinical endpoints.

An important observation is that HRV indices showed distinct associations with cardiac involvement and overall disease severity. Although several HRV measures were lower in patients with CMR-confirmed cardiac involvement, these associations did not remain independent after adjustment for troponin, suggesting that HRV should not be interpreted as a surrogate of myocardial injury or a replacement for established cardiac biomarkers when assessing cardiac involvement. By contrast, long-term HRV—particularly SDANN—remained independently associated with Mayo stage, a composite indicator of systemic disease burden. Together, these findings imply that HRV reflects an integrated physiological signal that likely captures the combined effects of myocardial involvement (e.g., altered ventricular mechanics and neurohormonal activation) and broader autonomic/systemic factors, rather than myocardial amyloid burden alone.

Several mechanisms have been proposed in the literature to account for observations of this kind; none was tested in the present study, which included neither neural histology nor dedicated autonomic reflex testing. Autonomic dysfunction in AL amyloidosis has been attributed to amyloid deposition in peripheral autonomic nerves and cardiac neural structures, with consequent impairment of sympathetic and parasympathetic signalling and of baroreflex and cardiovagal control. In addition, systemic inflammation, oxidative stress, and chronic organ dysfunction may further disturb central and peripheral autonomic regulation, thereby contributing to sustained autonomic imbalance. Structural cardiac remodeling and impaired ventricular compliance in advanced cardiac amyloidosis can also alter hemodynamic loading conditions and reflex arcs, resulting in secondary changes in autonomic tone and attenuation of long-term HRV components. These mechanisms are not mutually exclusive, and the present data cannot adjudicate among them or exclude a purely secondary origin for the reduction in long-term autonomic variability seen at more advanced disease stages (26).

We also explored the short-term effects of plasma cell–directed therapy on autonomic function. Among patients who underwent repeat Holter monitoring after at least three months of therapy (median interval 9.50 months), most HRV parameters did not change significantly. Because hematologic response was not evaluated separately within this paired subgroup, these data describe HRV before and after treatment rather than an association between HRV change and depth of hematologic response. The observation is compatible with persistence of autonomic dysfunction over this interval, but the limited sample size, heterogeneity of treatment regimens, and variable follow-up duration preclude any inference about the time course of autonomic recovery relative to clonal control. Longer-term, prospective studies are needed to determine whether sustained hematologic and organ responses, or specific therapeutic strategies, can ultimately lead to meaningful improvement in autonomic function. Of note, patients who completed repeat monitoring differed from those who did not in two baseline characteristics: SDNN was significantly higher in the follow-up group (*P* = 0.004) and NT-proBNP was significantly higher in the non-follow-up group (*P* = 0.024), indicating that the follow-up subset may represent a somewhat more clinically stable or adherent subset of the overall cohort; this potential selection bias should be considered when interpreting the treatment-response findings. Accordingly, the absence of measurable HRV recovery should be interpreted as a preliminary, hypothesis-generating observation rather than evidence that autonomic function is fixed. Two mechanisms plausibly bias this subgroup. Patients who died or deteriorated before the scheduled reassessment could not contribute a repeat recording, so the follow-up subset necessarily comprises survivors, which may have shifted the observed post-treatment HRV values upward. In addition, because repeat monitoring was ordered at the discretion of the treating physician rather than by protocol, indication bias cannot be separated from the treatment effect. Neither source of bias could be formally adjusted for in the present design.

From a clinical perspective, our findings suggest that 24 h HRV may serve as a simple, non-invasive adjunct for assessing autonomic modulation and disease severity in AL amyloidosis. In particular, reduced SDANN provided information complementary to cardiac biomarkers in characterising disease severity. However, HRV should be interpreted alongside established biomarkers and imaging findings and should not be used as a stand-alone tool for individual prognostic decision-making; normative HRV values vary substantially by age, sex, recording duration, and analytic methodology, and there is no universally accepted cutoff for individual-level prognostication. Although SDNN ≤50 ms has been reported to predict short-term mortality in a small AL amyloidosis cohort (20), externally validated, disease-specific thresholds are not yet available.

Several features of the study design warrant cautious interpretation. The retrospective, single-center design and the modest sample size, particularly the paired follow-up subgroup (*n* = 33), limit generalizability and statistical power, and no *a priori* power calculation was performed. Twenty-four-hour Holter monitoring characterises autonomic modulation but does not directly assess autonomic reflex function, and stage-stratified comparisons with healthy controls were not available. Disease duration at the time of HRV assessment was not reliably documented, because evaluations were performed at diagnosis or treatment initiation, so a disease-duration variable could not be included. We also did not systematically record acute stress, physical activity, caffeine intake, or other time-varying exposures at the time of Holter monitoring, all of which may influence HRV and may have introduced residual confounding. A further caution concerns beta-blocker therapy: SDNN was higher, not lower, in beta-blocker users, which is the opposite of the direction expected from rate control alone and most plausibly reflects confounding by indication, since patients with the most advanced cardiac involvement were least likely to tolerate these agents. Beta-blocker use was also absent in the control group by design, so the comparison between patients and controls could not be adjusted for it. Finally, because Mayo staging was analysed separately, this variable was not included in the baseline characteristics comparison.

The choice of control group deserves separate comment. Using healthy rather than disease controls limits our ability to separate amyloid-specific autonomic dysfunction from secondary autonomic dysregulation attributable to heart failure hemodynamics in general. However, our observation that 30.8% of CMR-negative patients already exhibited HRV abnormalities suggests that autonomic impairment may precede overt structural cardiac involvement. This provides physiological rationale that the reduced HRV in AL amyloidosis is partially driven by direct early amyloidogenic toxicity or infiltration into the autonomic nervous system, rather than being solely a secondary consequence of severe cardiac dysfunction.

Statistical constraints also temper these findings. Events-per-variable ratios in both multivariable models were modest, no formal correction for multiple testing was applied, and all reported *P* values should therefore be interpreted as nominal. The HRV findings in this study are hypothesis-generating and require confirmation in adequately powered prospective cohorts before clinical application. One further interpretive caveat applies specifically to the ordinal analysis. Because the Mayo 2004 stage is itself defined by cardiac troponin T and NT-proBNP, the association between hs-cTnT and stage partly reflects this definitional overlap rather than independent prediction, and should not be read as a discovery. The association of SDANN with stage is not subject to this circularity, since HRV indices do not enter the staging algorithm.

Beyond these design considerations, two methodological limitations warrant acknowledgment. First, mass spectrometry–based proteomic amyloid typing, which offers definitive fibril-protein identification, was not routinely available at our center during the study period; subtyping relied on immunohistochemical and immunofluorescence staining in the context of consistent hematologic and biochemical findings. This approach is widely used and is generally reliable when clinical, laboratory, and pathologic criteria are congruent, but the absence of mass spectrometry–based confirmation means that rare misclassification of non-AL subtypes cannot be entirely excluded. Second, although all recordings were acquired and processed on the same TLC6000 system using the manufacturer's default filtering and windowing settings, the specific version of the proprietary analysis software was not systematically recorded in the clinical database during the study period, which constrains exact reproduction of the processing pipeline by other groups.

Two further limitations relate to data collection. First, although both Holter monitoring and CMR were performed at baseline near the time of diagnosis, the interval between the two assessments was not systematically recorded in the clinical database; residual inter-individual variability in their timing therefore cannot be quantified and may have introduced noise in the observed associations. Second, treatment regimens were not documented at the individual-patient level in a form that allowed patients to be grouped by regimen, and the follow-up subgroup was small (*n* = 33); formal stratification of HRV trajectories by treatment type was therefore not feasible. Whether different plasma cell-directed regimens differentially affect autonomic recovery remains an important unanswered question that warrants investigation in adequately powered prospective cohorts. Larger prospective cohorts are needed to validate individual-level cutoffs and to establish the stage-specific screening value of HRV.

## 5 Conclusion

Our study demonstrates that autonomic dysfunction, as reflected by reduced HRV, is a prominent feature of AL amyloidosis. Beyond merely reflecting the presence of cardiac involvement, reduced long-term HRV (SDANN) independently associated with advanced disease stages, providing information on disease severity that complements established cardiac biomarkers. These findings support the concept that autonomic dysfunction represents a distinct and clinically relevant dimension of disease severity in AL amyloidosis. Whether HRV can provide independent prognostic value beyond established biomarkers—and ultimately aid clinical risk stratification—remains to be established; the present study did not evaluate clinical endpoints such as mortality or hospitalization, and formal evaluation in outcome-powered prospective cohorts is required before clinical utility conclusions can be drawn.

## Data Availability

The original contributions presented in the study are included in the article/Supplementary Material, further inquiries can be directed to the corresponding author.
