# Supplementary material for: Reduced heart rate variability is associated with disease severity in AL amyloidosis: a retrospective cohort study
Source: Front Med (Lausanne). 2026 Aug 27;13:1882210. doi: 10.3389/fmed.2026.1882210 (PMC13558003; doi:10.3389/fmed.2026.1882210)
Supplement: Supplementary file 1 [file Table1.docx]

Supplementary Table S1. Effect of medication on HRV in patients with AL amyloidosis.

|  | β-blocker (-) (*n*=42) | β-blocker (+) (*n*=14) | *P* | ACEI/ARB (-) (*n*=40) | ACEI/ARB (+) (*n*=16) | *P* |
| --- | --- | --- | --- | --- | --- | --- |
| Mean HR (bpm) | 80.21±10.12 | 68.64±5.67 | <0.001 | 77.03±10.98 | 78.06±9.33 | 0.741 |
| SDNN(ms) | 86.11±30.74 | 111.19±41.31 | 0.019 | 90.71±31.83 | 96.56±42.90 | 0.578 |
| SDANN(ms) | 111.20±47.85 | 119.61±36.65 | 0.551 | 118.36±47.86 | 100.67±35.73 | 0.188 |
| rMSSD(ms) | 29.25(18.68-46.10) | 27.30(21.10-79.35) | 0.670 | 28.05 (16.28-66.78) | 28.70(22.60-39.25) | 0.724 |
| pNN50(%) | 2.60(0.90-5.35) | 2.60(0.75-20.13) | 0.740 | 3.35 (0.85-9.10) | 2.05(0.90-2.88) | 0.360 |
| CV | 0.04±0.03 | 0.05±0.04 | 0.398 | 0.05±0.03 | 0.04±0.03 | 0.527 |
| TP (ms^2^) | 4967.00(1953.90-5419.20) | 5486.50(2946.35-7372.15) | 0.154 | 5004.85 (2386.98-5470.93) | 4653.00(2193.18-7344.78) | 0.609 |
| ULF(ms^2^) | 2274.80(1130.10-4453.90) | 2832.00(1735.10-5700.95) | 0.172 | 2318.30(1242.75-4481.38) | 3006.85(1457.35-5684.60) | 0.495 |
| VLF(ms^2^) | 665.70(276.85-1077.45) | 848.60(461.45-1353.85) | 0.270 | 623.55(342.75-1221.80) | 773.70(187.93-881.90) | 0.570 |
| LF(ms^2^) | 72.90(34.80-215.95) | 51.20(41.60-250.65) | 0.944 | 75.20(36.73-239.93) | 65.10(32.98-126.63) | 0.747 |
| HF(ms^2^) | 70.80(25.45-188.05) | 54.10 (32.85-336.70) | 0.785 | 67.05(18.58-293.25) | 67.90(40.20-150.53) | 0.705 |

Abbreviations: HRV, heart rate variability; AL, light chain amyloidosis; ACEI, angiotensin-converting enzyme inhibitors; ARB, angiotensin receptor blockers; SDNN, standard deviation of all normal-to-normal RR intervals; SDANN, standard deviation of the average normal-to-normal RR intervals for all 5-minute segments; rMSSD, root mean square of successive differences between adjacent normal RR intervals; pNN50, percentage of successive normal RR intervals differing by more than 50 ms; CV, coefficient of variation; TP, total power; ULF, ultra-low frequency; VLF, very-low frequency; LF, low frequency; HF, high frequency.
