# Supplementary material for: Reduced heart rate variability is associated with disease severity in AL amyloidosis: a retrospective cohort study
Source: Front Med (Lausanne). 2026 Aug 27;13:1882210. doi: 10.3389/fmed.2026.1882210 (PMC13558003; doi:10.3389/fmed.2026.1882210)
Supplement: Supplementary file 2 [file Table2.docx]

Supplementary Table S2. Events-per-variable (EPV) analysis and complete regression outputs for multivariable models.

Section A. Events-per-variable analysis — CMR-positive cardiac involvement (binary logistic regression)

| Model Component | Value | Threshold | Interpretation |
| --- | --- | --- | --- |
| Total sample (N) | 56 | — | — |
| CMR-positive (events, outcome = 1) | 43 | — | — |
| CMR-negative (non-events, outcome = 0) | 13 | — | — |
| Limiting class (minority class) | 13 (CMR-negative) | — | EPV denominator |
| Candidate variables (univariable *P* < 0.05) | 5 (log NT-proBNP, log hs-cTnT, SDANN, ULF, sex) | — | — |
| EPV if all 5 entered simultaneously | 13 / 5 = 2.6 | ≥ 10 (conventional) | Below threshold → overfitting risk |
| Variables in final multivariable model | 1 (log hs-cTnT, backward stepwise) | — | EPV = 13 → acceptable |
| EPV in final model | 13 / 1 = 13.0 | ≥ 10 | Above threshold (EPV = 13.0) |
| VIF (all candidate predictors) | All < 3.0 | < 10 (no collinearity) | VIF within acceptable range |
| LASSO (sensitivity) | log hs-cTnT, log NT-proBNP, ULF, sex (non-zero) | — | Concordant with stepwise model |

Section B. Events-per-variable analysis — Mayo 2004 disease stage (ordinal logistic regression)

| Model Component | Value | Threshold | Interpretation |
| --- | --- | --- | --- |
| Total sample (N) | 56 | — | — |
| Stage I / II / III (n) | 11 / 20 / 25 | — | — |
| Minority class (Stage I) | n = 11 | — | EPV denominator |
| Candidate variables (univariable *P* < 0.05) | SDANN, hs-cTnT (and others) | — | — |
| Final model variables (stepwise) | 2 (SDANN, hs-cTnT) | — | EPV = 11 / 2 = 5.5 |
| EPV in final model | 11 / 2 = 5.5 | ≥ 10 (conventional) | Below ideal; results interpreted cautiously |
| Proportional-odds assumption | Test of parallel lines: *P* = 0.571 | *P* > 0.05 → not violated | Proportional-odds assumption not violated |
| VIF (SDANN, hs-cTnT) | < 3.0 | < 10 | VIF within acceptable range |

Section C. Complete regression outputs — CMR-positive cardiac involvement (binary logistic regression; each potential confounder entered individually)

| Model | Variable | β | SE | OR | 95% CI | *P* |
| --- | --- | --- | --- | --- | --- | --- |
| Base model (ln hs-cTnT alone) | ln hs-cTnT | 1.973 | 0.543 | 7.19 | 2.48–20.84 | <0.001 |
| + Beta-blocker use | ln hs-cTnT | 2.107 | 0.639 | 8.22 | 2.35–28.76 | 0.001 |
|  | Beta-blocker use | -0.736 | 1.383 | 0.48 | 0.03–7.21 | 0.595 |
| + Mean heart rate | ln hs-cTnT | 2.152 | 0.623 | 8.60 | 2.54–29.17 | <0.001 |
|  | Mean heart rate | 0.054 | 0.054 | 1.06 | 0.95–1.18 | 0.318 |
| + Diabetes | ln hs-cTnT | 1.921 | 0.540 | 6.83 | 2.37–19.67 | <0.001 |
|  | Diabetes | -1.121 | 1.562 | 0.33 | 0.02–6.96 | 0.473 |
| + Hypertension | ln hs-cTnT | 2.278 | 0.726 | 9.76 | 2.35–40.51 | 0.002 |
|  | Hypertension | -1.263 | 1.234 | 0.28 | 0.03–3.18 | 0.306 |
| + ACEI/ARB use | ln hs-cTnT | 2.008 | 0.570 | 7.45 | 2.44–22.78 | <0.001 |
|  | ACEI/ARB use | -0.298 | 1.045 | 0.74 | 0.10–5.75 | 0.776 |

Section D. Complete regression outputs — Mayo 2004 disease stage (ordinal logistic regression; each potential confounder entered individually)

| Model | Variable | β | SE | OR | 95% CI | *P* |
| --- | --- | --- | --- | --- | --- | --- |
| Base model (SDANN + hs-cTnT) | SDANN | -0.016 | 0.007 | 0.985 | 0.972–0.998 | 0.021 |
|  | hs-cTnT | 0.043 | 0.015 | 1.044 | 1.015–1.075 | 0.003 |
| + Beta-blocker use | SDANN | -0.015 | 0.007 | 0.985 | 0.972–0.998 | 0.024 |
|  | hs-cTnT | 0.044 | 0.015 | 1.045 | 1.015–1.075 | 0.003 |
|  | Beta-blocker use | -0.305 | 0.630 | 0.74 | 0.21–2.53 | 0.628 |
| + Mean heart rate | SDANN | -0.012 | 0.007 | 0.988 | 0.974–1.002 | 0.092 |
|  | hs-cTnT | 0.046 | 0.015 | 1.047 | 1.017–1.078 | 0.002 |
|  | Mean heart rate | 0.039 | 0.032 | 1.04 | 0.98–1.11 | 0.229 |
| + Diabetes | SDANN | -0.017 | 0.007 | 0.984 | 0.970–0.997 | 0.016 |
|  | hs-cTnT | 0.046 | 0.016 | 1.048 | 1.016–1.080 | 0.003 |
|  | Diabetes | 1.190 | 0.874 | 3.29 | 0.59–18.23 | 0.174 |
| + Hypertension | SDANN | -0.014 | 0.007 | 0.986 | 0.973–1.000 | 0.044 |
|  | hs-cTnT | 0.047 | 0.016 | 1.048 | 1.016–1.082 | 0.004 |
|  | Hypertension | 1.099 | 0.615 | 3.00 | 0.90–10.02 | 0.074 |
| + ACEI/ARB use | SDANN | -0.018 | 0.007 | 0.982 | 0.968–0.996 | 0.011 |
|  | hs-cTnT | 0.040 | 0.015 | 1.041 | 1.011–1.072 | 0.007 |
|  | ACEI/ARB use | -0.764 | 0.646 | 0.47 | 0.13–1.65 | 0.237 |

Section E. Sensitivity analysis excluding all 14 beta-blocker users (n = 42)

| Model | Variable | β | SE | OR | 95% CI | *P* |
| --- | --- | --- | --- | --- | --- | --- |
| CMR positivity (n = 42) | ln hs-cTnT | 1.835 | 0.597 | 6.27 | 1.95–20.18 | 0.002 |
| Mayo stage (n = 42) | SDANN | -0.015 | 0.008 | 0.985 | 0.971–1.000 | 0.053 |
|  | hs-cTnT | 0.047 | 0.019 | 1.048 | 1.010–1.087 | 0.012 |

Because only 13 CMR-negative events were available, each potential confounder was entered individually into the base model rather than simultaneously, in accordance with events-per-variable principles (Section A). In the Mayo ordinal model, EPV was 5.5 (Section B)—below the conventional threshold of 10 but above the minimum acceptable level; two predictors were retained because both were pre-specified on pathophysiological grounds and the proportional-odds assumption was satisfied (test of parallel lines: *P* = 0.571). In the sensitivity analysis excluding all 14 beta-blocker users (Section E, n = 42), the SDANN association attenuated to borderline significance (*P* = 0.053), consistent with reduced statistical power in the smaller sample; the hs-cTnT associations for both outcomes remained statistically significant. hs-cTnT was natural-log transformed in the CMR models and analysed on the original scale in the ordinal models. β denotes the regression coefficient and SE its standard error; ORs for continuous predictors correspond to a one-unit increase.

Abbreviations: CMR, cardiac magnetic resonance; EPV, events per variable; VIF, variance inflation factor; LASSO, least absolute shrinkage and selection operator; log / ln, natural-log transformed; hs-cTnT, high-sensitivity cardiac troponin T; NT-proBNP, N-terminal pro-B-type natriuretic peptide; SDANN, standard deviation of the average normal-to-normal intervals for successive 5-minute segments; ULF, ultra-low frequency; ACEI, angiotensin-converting enzyme inhibitor; ARB, angiotensin receptor blocker; SE, standard error; OR, odds ratio; CI, confidence interval.
