# Supplementary material for: Reduced heart rate variability is associated with disease severity in AL amyloidosis: a retrospective cohort study
Source: Front Med (Lausanne). 2026 Aug 27;13:1882210. doi: 10.3389/fmed.2026.1882210 (PMC13558003; doi:10.3389/fmed.2026.1882210)
Supplement: Supplementary file 3 [file Table3.docx]

Supplementary Table S3. Baseline characteristics of patients with and without follow-up Holter recordings.

| Variable | Follow-up group (n = 33) | No follow-up (n = 23) | *P* |
| --- | --- | --- | --- |
| Age (years) | 63.39 ± 8.36 | 61.65 ± 10.25 | 0.887 |
| Sex (male), n (%) | 17 (51.5) | 12 (52.2) | 1.000 |
| Mayo 2004 stage (I/II/III), n | 8 / 11 / 14 | 3 / 9 / 11 | 0.582 |
| NT-proBNP (pg/mL) | 2408.94 ± 2591.10 | 5882.13 ± 6724.77 | 0.024* |
| hs-cTnT (ng/L) | 37.89 ± 58.57 | 42.33 ± 47.71 | 0.286 |
| Mean heart rate (bpm) | 74.64 ± 8.03 | 81.17 ± 12.39 | 0.064 |
| SDNN (ms) | 101.48 ± 29.61 | 77.81 ± 38.23 | 0.004** |
| SDANN (ms) | 114.71 ± 39.58 | 111.84 ± 52.97 | 0.405 |

Values are mean ± SD unless otherwise noted. Because both subgroups were small, all continuous variables were compared with the Mann-Whitney U test rather than selecting between parametric and non-parametric tests on a variable-by-variable basis; means and standard deviations are shown for comparability with Table 1. Categorical variables were compared with the chi-square or Fisher's exact test. * *P* < 0.05; ** *P* < 0.01.

Abbreviations: hs-cTnT, high-sensitivity cardiac troponin T; NT-proBNP, N-terminal pro-B-type natriuretic peptide; SDNN, standard deviation of all normal-to-normal RR intervals; SDANN, standard deviation of the average normal-to-normal intervals for successive 5-minute segments.
